# Supplementary material for: AI-guided transition path sampling of lipid flip-flop and membrane nanoporation
Source: Nat Commun. 2025 Dec 22;17:224. doi: 10.1038/s41467-025-67599-3 (PMC12780044; doi:10.1038/s41467-025-67599-3)
Supplement: Supplementary file 1 — Supplementary Information [file 41467_2025_67599_MOESM1_ESM.pdf]

AI-guided transition path sampling of lipid flip-flop and membrane nanoporation

Supplementary Information

Matthias Post and Gerhard Hummer*

Max Planck Institute of Biophysics, 60438 Frankfurt am Main, Germany

(Juli 28, 2025)

*email: gerhard.hummer@biophys.mpg.de

Supplementary Figures

TPS of Martini DMPC lipids

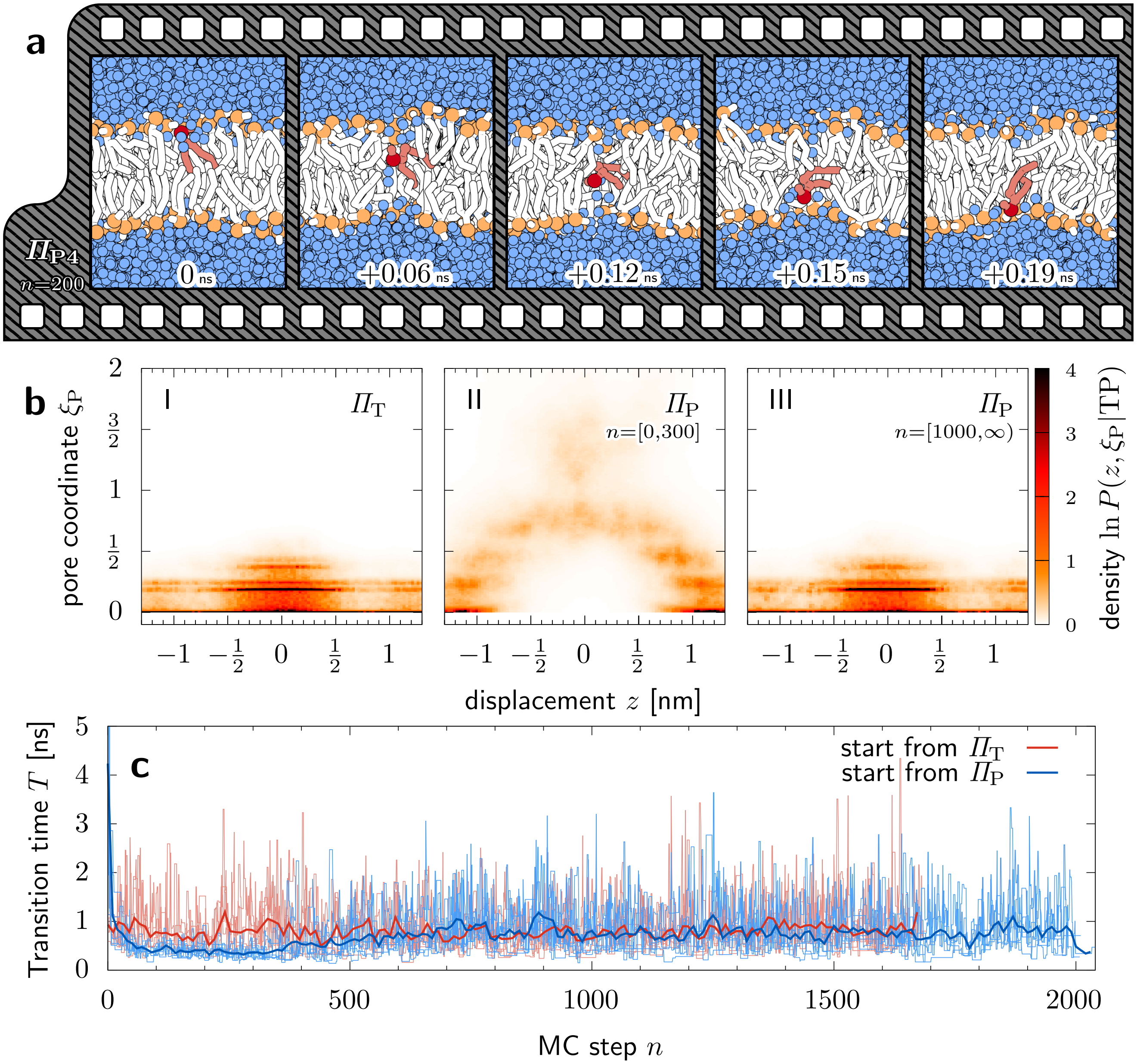

Supplementary Figure 1:. Intermediate flip-flop mechanism of Martini DMPC lipids. **a** Exemplary trajectory during the intermediate ($n \approx 200$) interval of one of the Π_P MC chains showing the pore collapse. Snapshots show representative intermediate conformations. Water beads in blue, lipids in white, PO4 beads orange, probe lipid in red. The box is sliced in half to see the inside. **b** k-nearest neighbor estimate of the TP density (with $k = 500$) along transversal displacement z and pore reaction coordinate ξ_P, adopted from Refs. 1,2. We compare the TPS MC chains starting in Π_T (bI), with those starting in Π_P, split into beginning ($n \in [0, 300]$, bII) and the end ($n \in [0, \infty)$, bIII) of the MC chain. **c** Transition times evolving with the MC chains, comparing tunnel (Π_T, red) and pore mechanism (Π_P, blue). Dark colors show an average over the faint samples, smoothed over 10 MC steps.

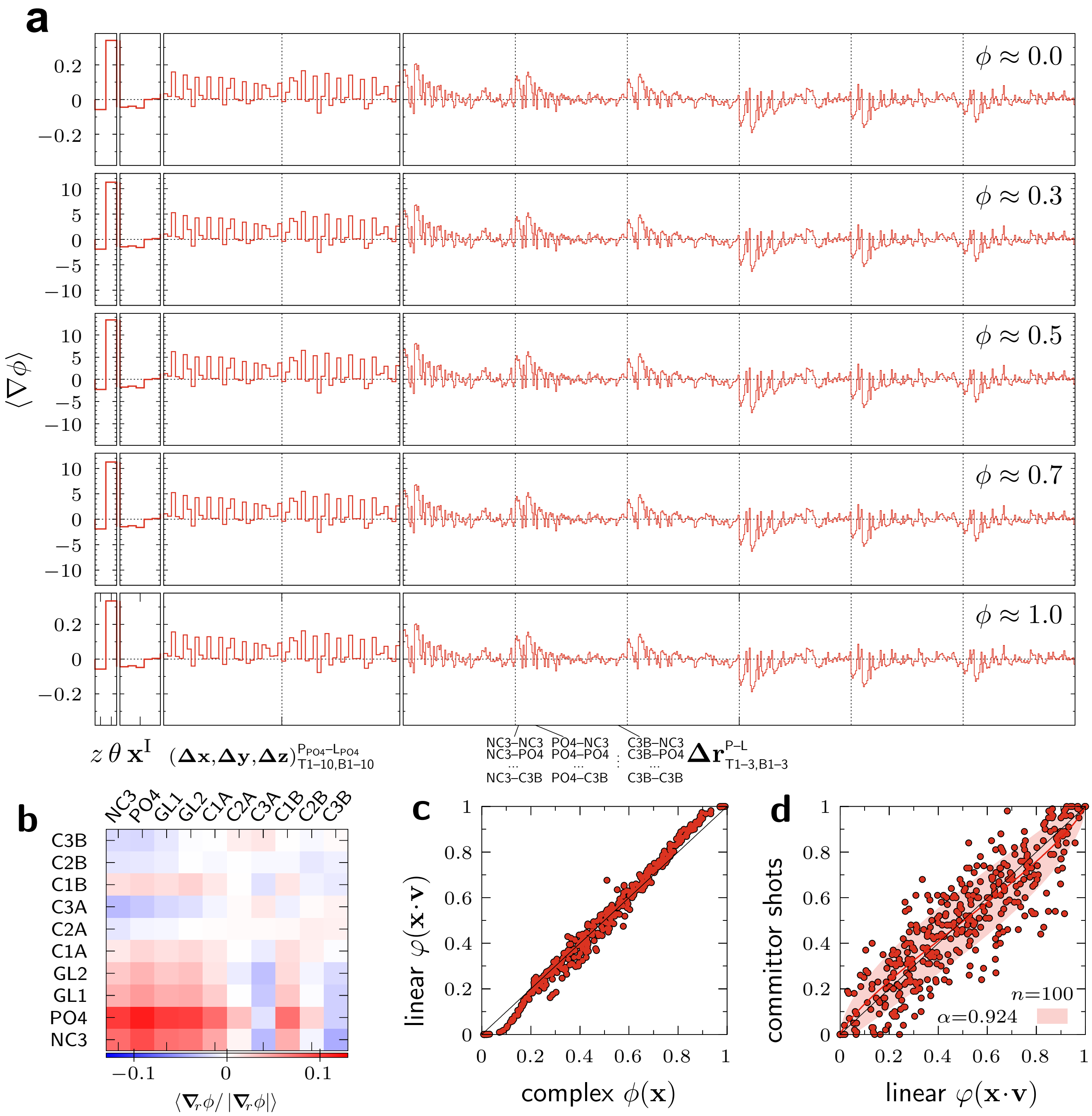

Supplementary Figure 2: Features of committor model for flip-flop of Martini DMPC lipids. **a** Average gradient of the committor conditioned to specific committor values. We extracted all structures of the transition path ensemble in the window $\phi \pm \Delta\phi$, with $\Delta\phi = 0.01$, and then averaged their $\nabla\phi$, as calculated from the network model. $\nabla\phi$ was further averaged over 10 models learned in each fold for cross validation. Features are grouped along the horizontal axis as indicated. **b** Normalized weights of the distances $\Delta\mathbf{r}^{\text{all}}$, averaged over all data and over upper and lower (inverted sign) leaflet neighbors. **c** Scatter plot comparing committor estimates of the neural network using all coordinates of the "final model" (Supplementary Table 4) with using only a linear combination of neighbor distances, $\Delta\mathbf{r}^{\text{all}} \cdot \mathbf{v}_{\text{r}}$, using conformations picked from the TPS data uniformly in ϕ. **d** Committor estimate of the same conformations, compared to the outcome of 100 shots from these conformations.

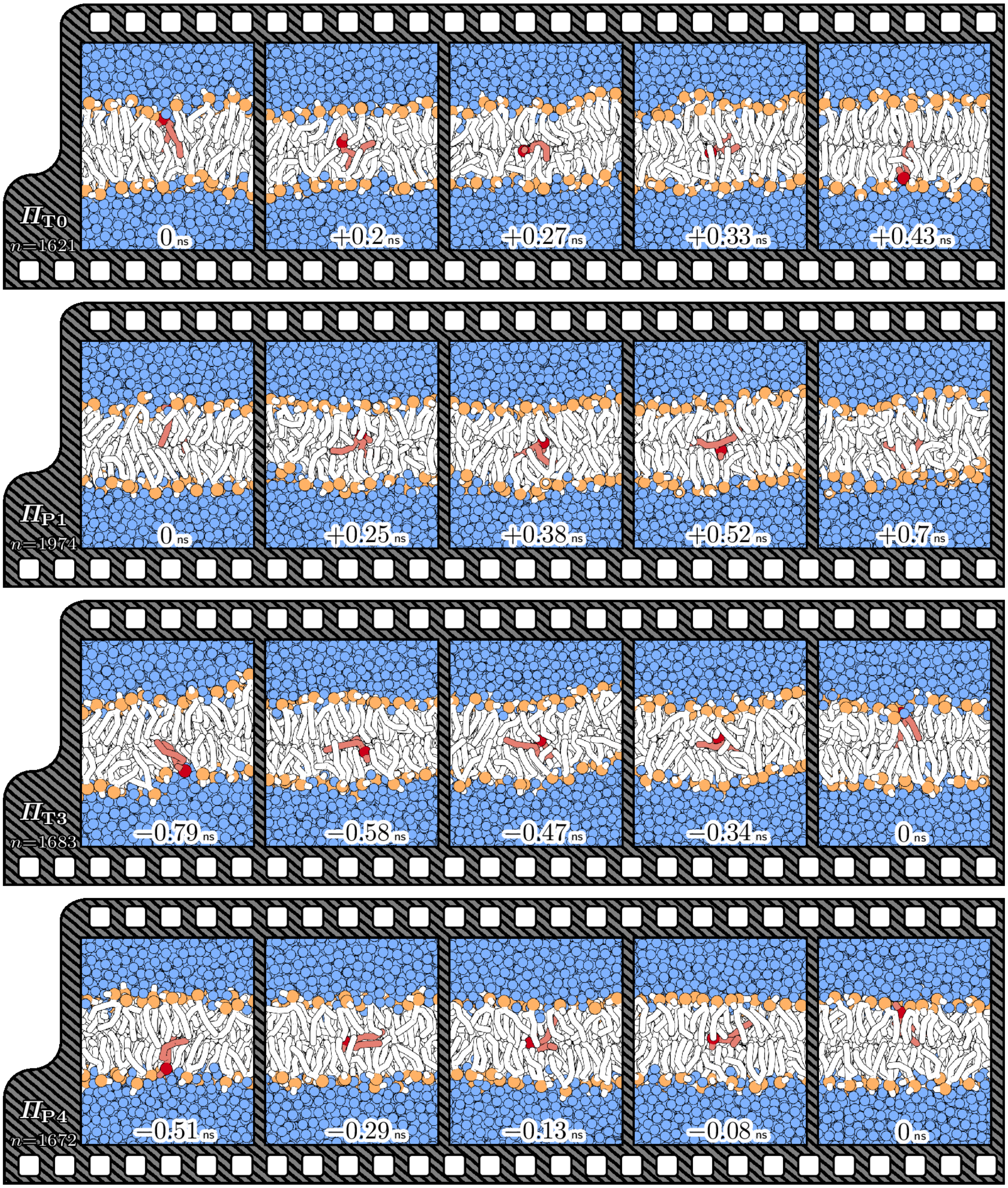

Supplementary Figure 3: Flip-flop mechanism of Martini DMPC lipids. Snapshots of exemplary flip-flop trajectories showing different intermediate configurations of the lipid probe during the dry Π_{T} transition. of two of the samplers traversing $\mathcal{U} \to \mathcal{L}$ from a 225/225 lipid distribution to 224/226, and two in the opposite $\mathcal{L} \to \mathcal{U}$ from 224/226 to 225/225, respectively. We pick frames close to the calculated committor values $\phi = 0.0$, 0.3, 0.5, 0.7 and 1.0, with time shown with white contour. Water and ion beads in blue. Water beads in blue, lipids in white, PO4 beads orange, probe lipid in red. The box is sliced in half to see the inside.

TPS of Charmm36 DMPC lipid flip-flop

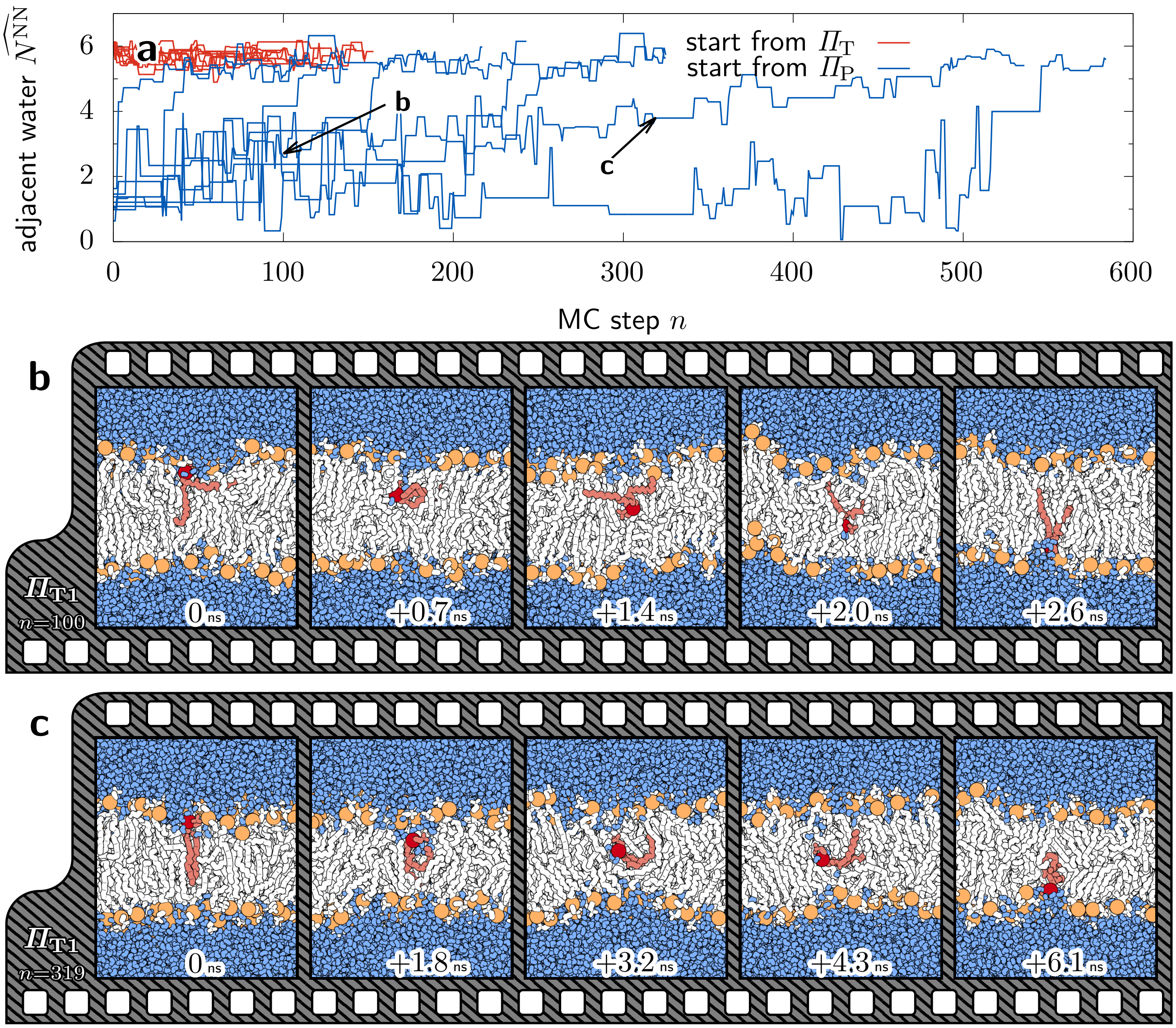

Supplementary Figure 4: Intermediate flip-flop mechanism of Charmm36 DMPC lipids. **a** Average number N^{NN} of water beads adjacent to lipid probe, as a function of TPS MC step, comparing samplers starting in the tunnel ($\mathit{\Pi}_\mathrm{T}$, red) and in the pore mechanism ($\mathit{\Pi}_\mathrm{P}$, blue). **b** Exemplary trajectory of a $\mathit{\Pi}_\mathrm{T}$ sampler at the start of the chain, where $\hat{\xi}_\mathrm{P} < 0.3$, dragging a few water molecules to the other side. **c** Exemplary transition during of the intermediate nucleation transition from $\mathit{\Pi}_\mathrm{T}$ to $\mathit{\Pi}_\mathrm{P}$, where $0.3 < \hat{\xi}_\mathrm{P} < 0.9$, showing the attempt of nucleating a pore during flip-flop. Snapshots show representative intermediate conformations. Water and ions in blue, lipids in white, probe lipid in red. The box is sliced in half to see the inside.

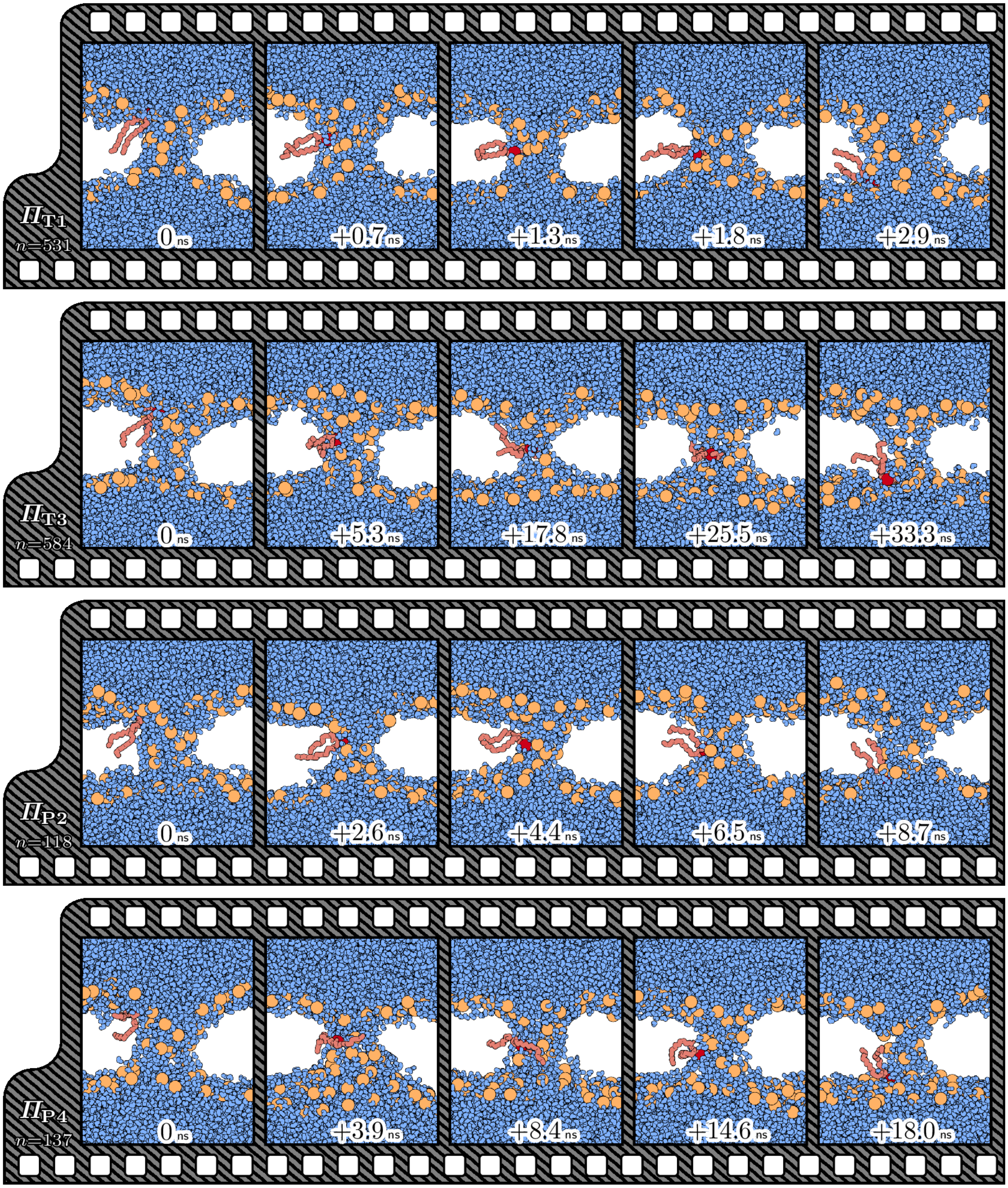

Supplementary Figure 5: Flip-flop mechanism of Charmm36 DMPC lipids. Snapshots of exemplary transition trajectories, from the last trajectories of two of the samplers starting in Π_{T} and Π_{P}, each, all showing the final diffusive pore-mediated flip-flop. We pick frames close to the committor values ϕ = 0.0, 0.3, 0.5, 0.7 and 1.0, not showing excursions of the lipid probe. Water and ions in blue, lipids in white, probe lipid in red. The box is sliced in half to see the inside.

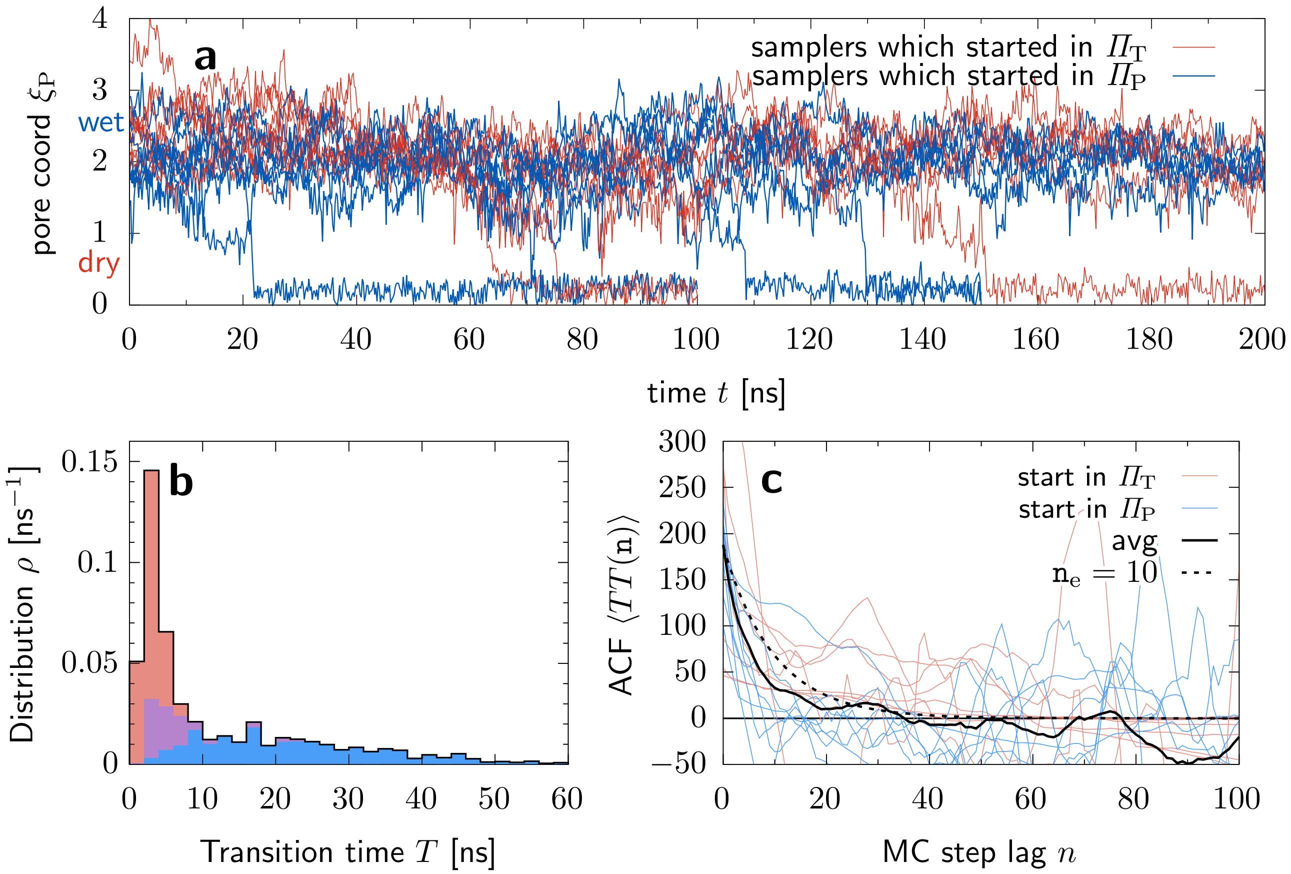

Supplementary Figure 6: Pore lifetime compared to flip-flop transition time. **a** Time evolution of unbiased simulations starting from shooting point structures of the last MC step. Simulations run either a maximum of 200 ns or until collapse of the pore. We measure the rate of pore closing by counting the fraction of trajectories which did finish before the 200 ns, and divided by the aggregate simulation time to get a maximum-likelihood estimate for the closing rate of about 2.326 μs^{-1} (Methods). In the simulations with open pores, lipids were flip-flopping with a rate of $\approx$ 93 μs^{-1}, suggesting around 15 lipid flip-flops during the pore lifetime. **b** Histogram of flip-flop transition time distribution, comparing trajectories utilizing Π_T (red), Π_P (blue), and the intermediate nucleation transition mechanism (violet). (**c**) Auto-correlation function (ACF) of transition times between samples along the Markov chain, showing the individual sampler, as well as the average ACF (black). A bootstrap average of exponential fit parameters suggests a decorrelation "time" of 10 ± 2 MC steps (black dashes).

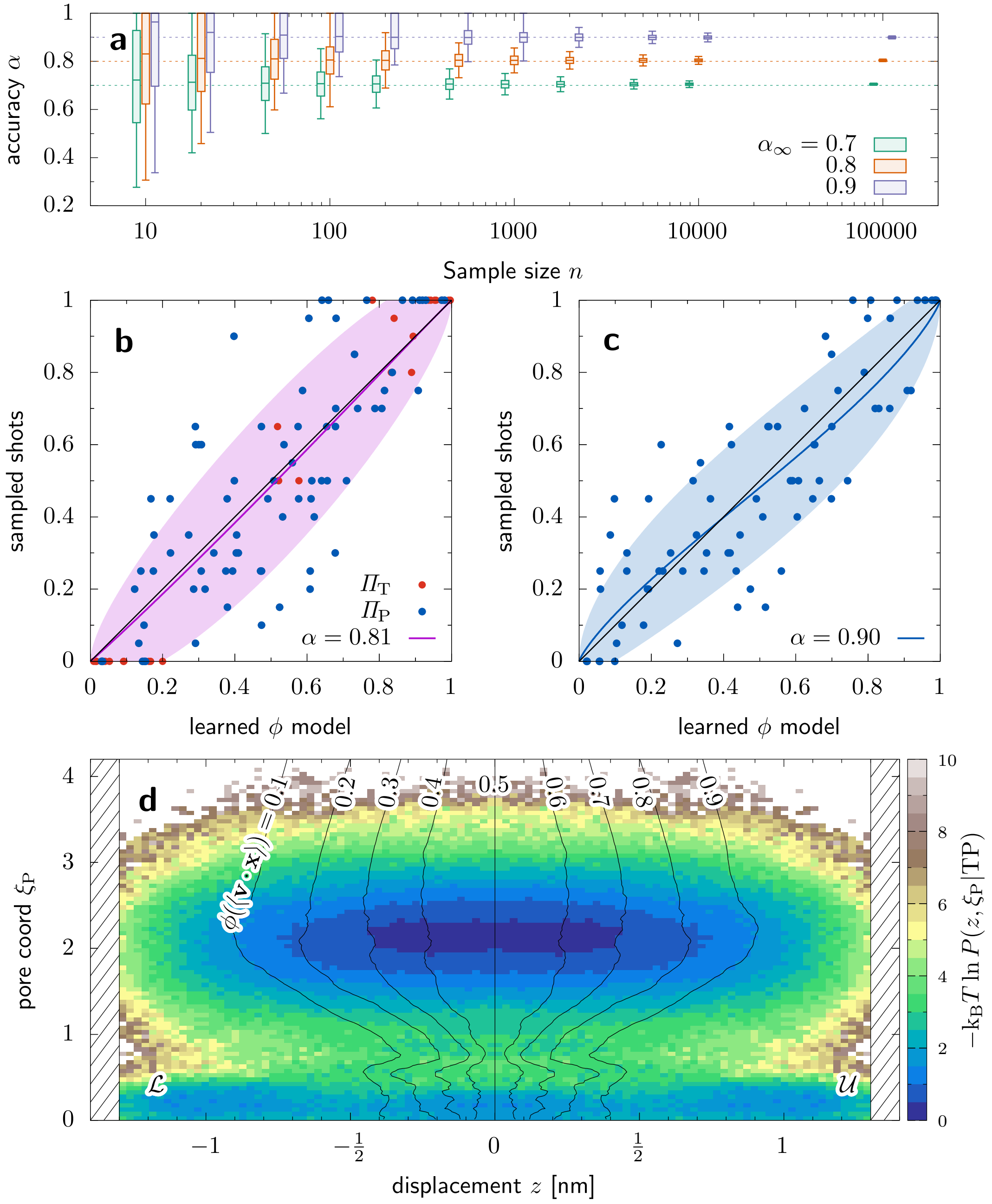

Supplementary Figure 7: Quality assessment of the Charmm36 DMPC TPE and committor estimate. a Evaluation of accuracy via synthetic data generated from n Bernoulli trials with probability logits drawn from a Cauchy (as done in AIMMD), and then adding noise to the probabilities as committor "estimates"; this is then repeated 1000 times and evaluated depicted as box plots, showing median, first and third quantile as box, 95% of data as whiskers. **b**,**c** Cross validation of all-atom committor model via committor shots. We picked at random 5 configurations close to $\phi_\mathrm{i} = 0.05 \times i$ and initiated 20 trajectory shots from each. **b** Model trained on whole data set, showing SPs resulting in a Π_T transition (red) and in a Π_P transition (blue). To identify possible systematic errors in the model, we transform the learned and sampled ϕ to the logits $q = \ln[\phi/(1-\phi)]$, and fit a linear ansatz function (see Methods of the main text). The error band of $\pm 1\sigma$ is then drawn (shading) according to the accuracy model (see Methods). **c** Model trained only on the Π_P data. **d** Symmetrized distribution of sampled transition along transversal displacement z and pore reaction coordinate ξ_P. We symmetrize by the mirror image at $z = 0$, both the density, here shown as a histogram, and the iso-lines of the committor (black lines) at the average feature vector parallel to the flux, $\mathbf{v} \cdot \mathbf{x}$.

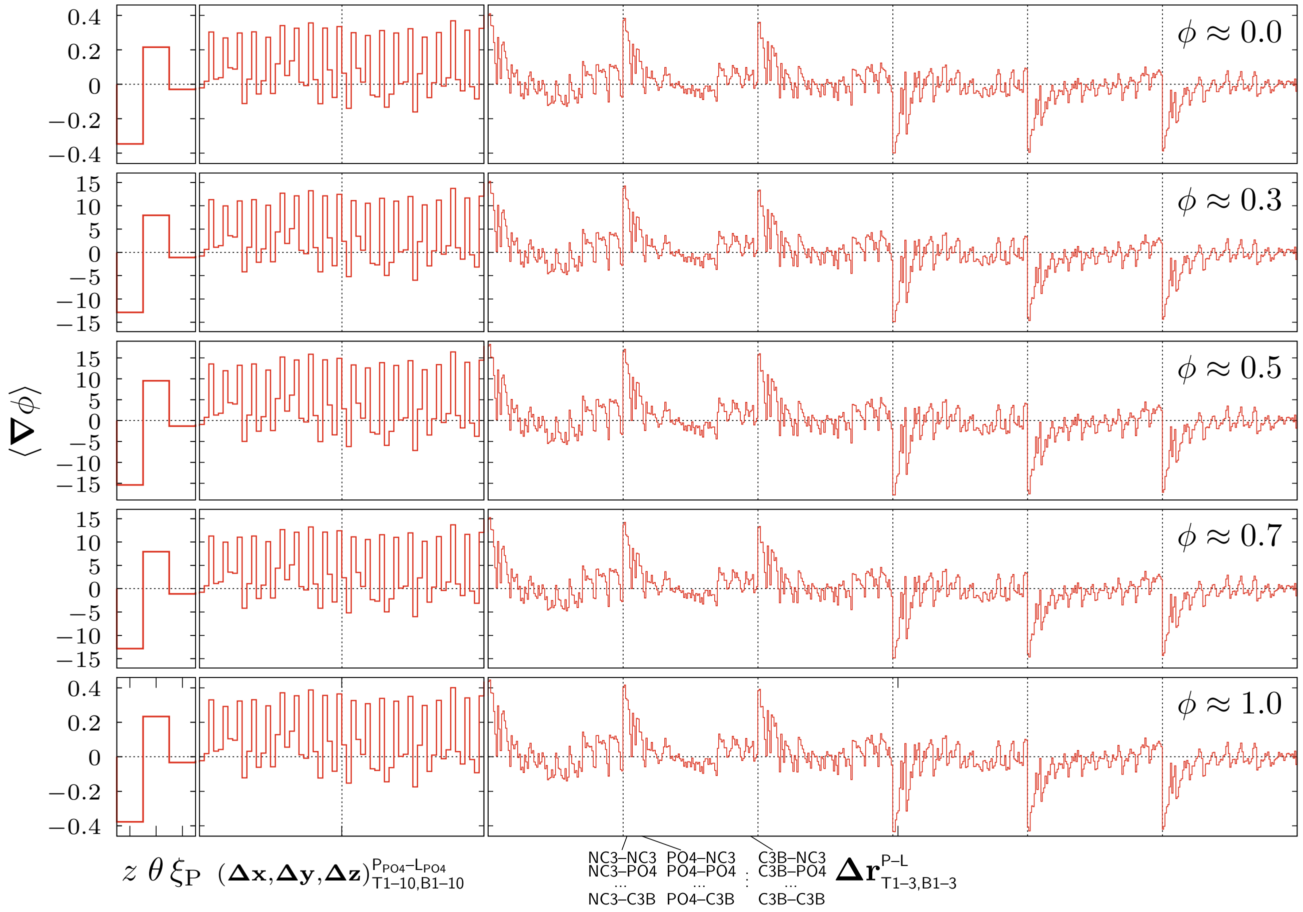

Supplementary Figure 8: Features of committor model in the all-atom DMPC lipid model. We compare the average gradient of the committor, conditioned to specific committor values. I.e., we extract all structures of the transition path ensemble in the window $\phi \pm \Delta\phi$, with $\Delta\phi = 0.01$, get $\nabla\phi$ from the network model, and then average over all frames of that window. $\nabla\phi$ was averaged over 10 models learned in each fold for cross validation.

TPS of Charmm36 DMPC pore nucleation

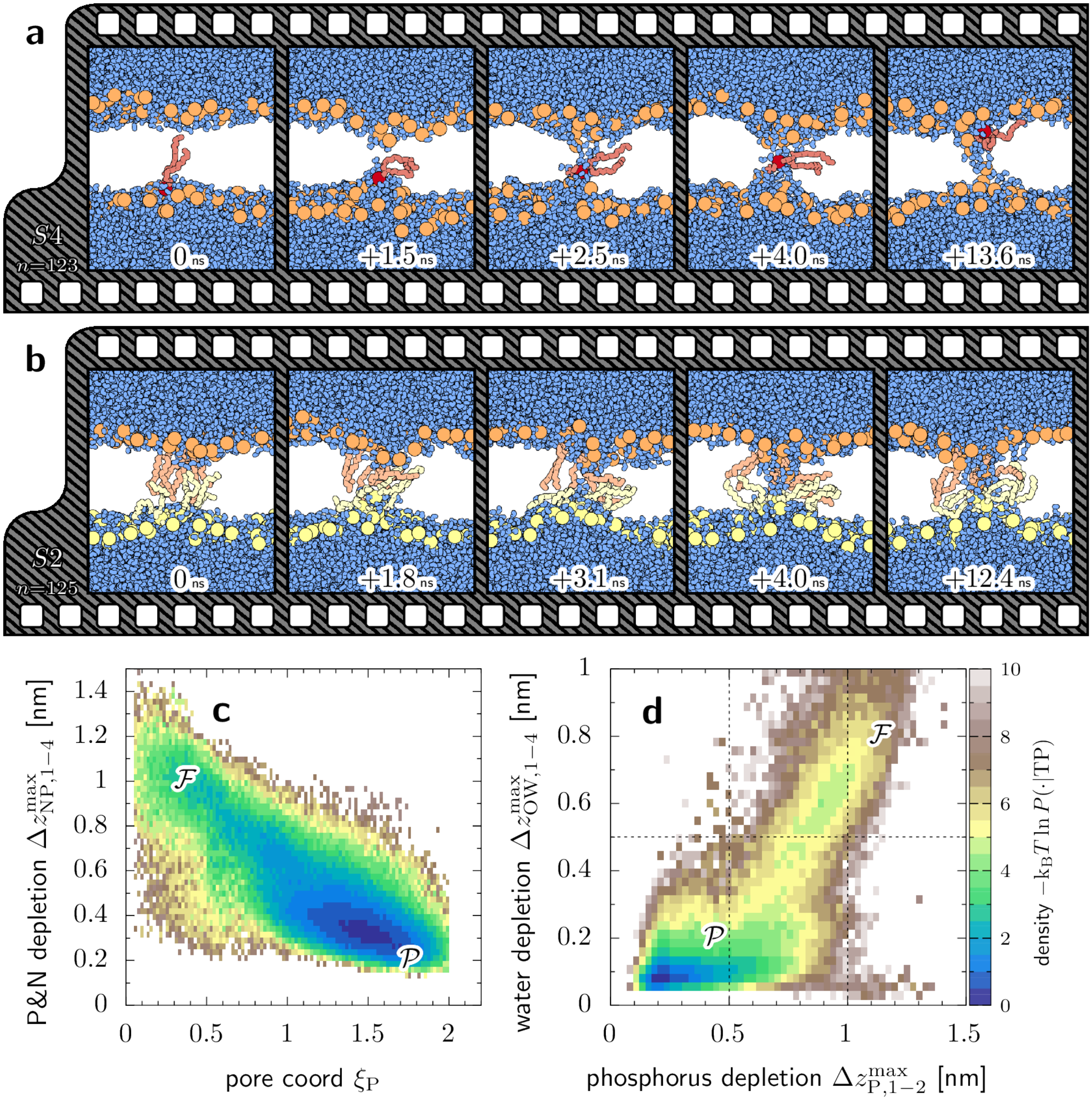

Supplementary Figure 9: Role of water and lipids during early pore nucleation. **a** Displacement of the lipid closest to the midplane (red) during pore nucleation, showing one rare occasion of successful flip-flop before the pore fully expanded. DMPC phosphorus atoms are shown in orange, water ions in blue. **b** Another nucleation TP, where no lipid from neither the upper (orange) nor the lower (yellow) leaflet is flipping in time. **c** Transition path ensemble projected onto pore reaction coordinate ξ_{P} from Refs. 1,2, compared to the largest depletion of phosphor and nitrogen, $\Delta z^{\max}_{\mathrm{NP},1-4}$, as described in Ref. 4. **d** Depletion of phosphorus, $\Delta z^{\max}_{\mathrm{P},1-2}$, compared to that of water, $\Delta z^{\max}_{\mathrm{OW},1-4}$, resolving two stages–water first, lipids second–of pore formation.

TPS of Charmm36 DSPC lipid flip-flop

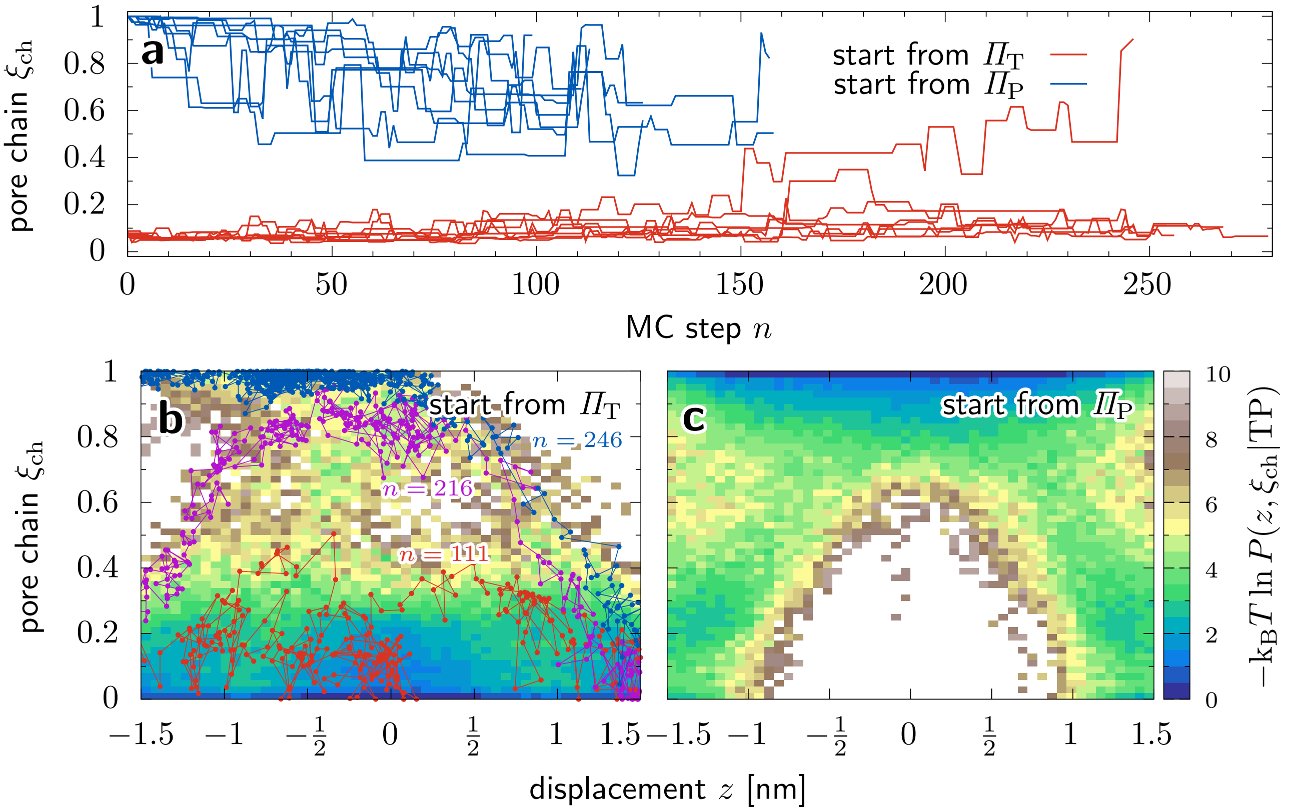

Supplementary Figure 10: DSPC lipid flip-flop. We use the same methodology as for the Charmm36 DMPC lipids to sample the TPE of DSPC membranes. **a** MC chains starting from a wet (Π_P) and a dry mechanism (Π_T), showing the time-averaged pore-chain coordinate $\hat{\xi}_{ch}$ of Ref. 1, measuring the fraction of the membrane at the nucleation center already occupied by water. **b**,**c** TPE projected onto the transversal displacement z and ξ_{ch} in TP samplers starting from Π_T **b** and Π_P **c**. In **b**, we highlight the trajectories of the sampler transitioning towards the pore mechanism with dots and lines. The depletion of the low-free energy region moving from the midplane ($z = 0$ nm, $\xi_{ch} \approx 1$, blue) towards one of the leaflets ($z = \pm 1.5$ nm) highlights that frequently the pore closes as soon the probed lipid finished flipping.

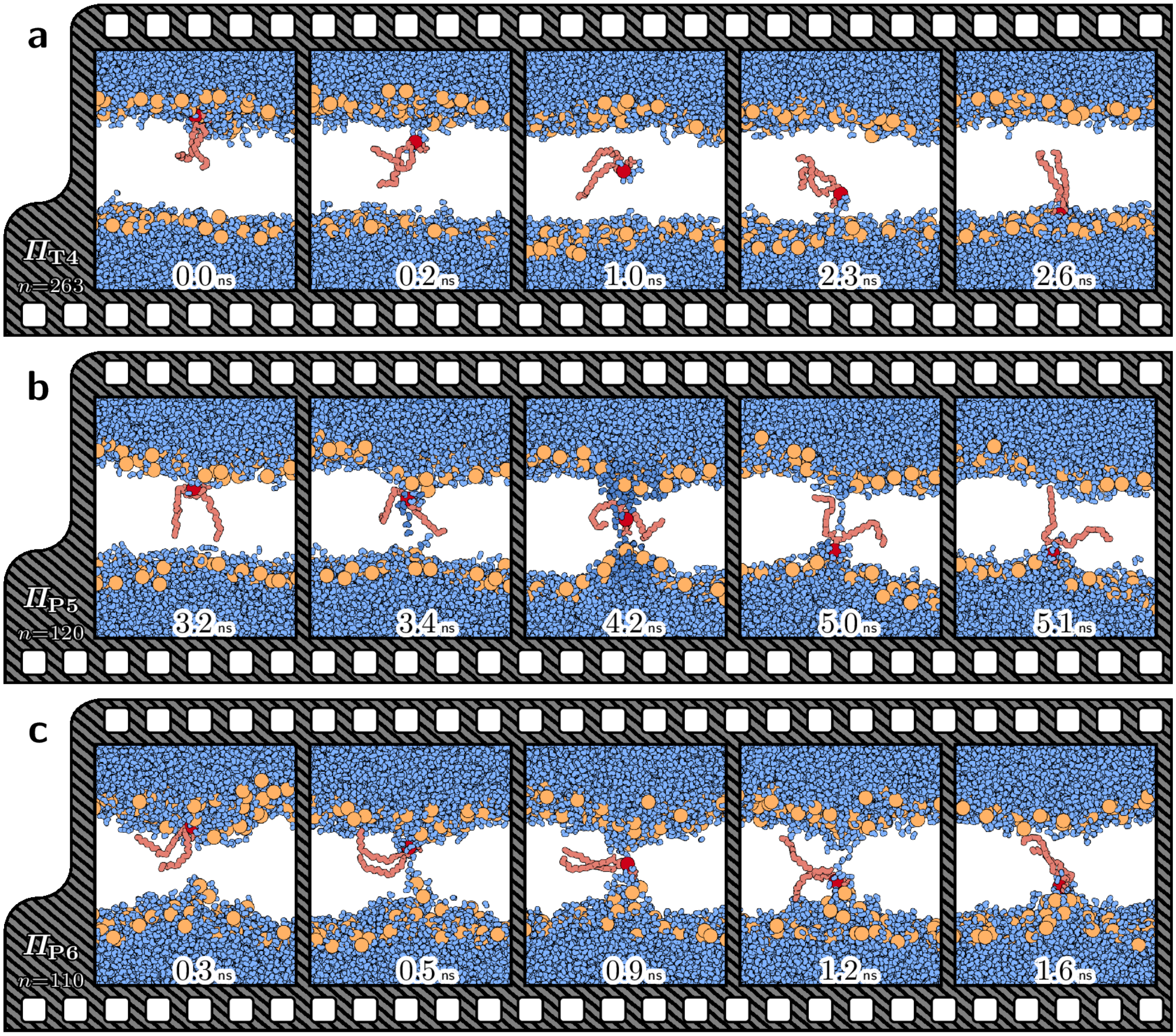

Supplementary Figure 11: Exemplary DSPC flip flop transitions. Lipid probe shown in red, DSPC phosphorus atoms are shown in orange, water ions in blue. **a** Lipid probe dragging water into the bilayer surrounding its head. **b** The formation of a water thread before and after the flip-flop event, with the formation of a cone-like structure at 4.2 ns. We color the water molecules close to the probe's head at that time in darker blue to indicate if and how fast the water molecules traverse the bilayer. **c** Another example of the dominant transition mechanism of samplers in the Π_P channel, showing how fast the pore is closing after flip-flop, as well as the initial local thinning.

TPS of mammalian plasma membrane lipid flip-flop

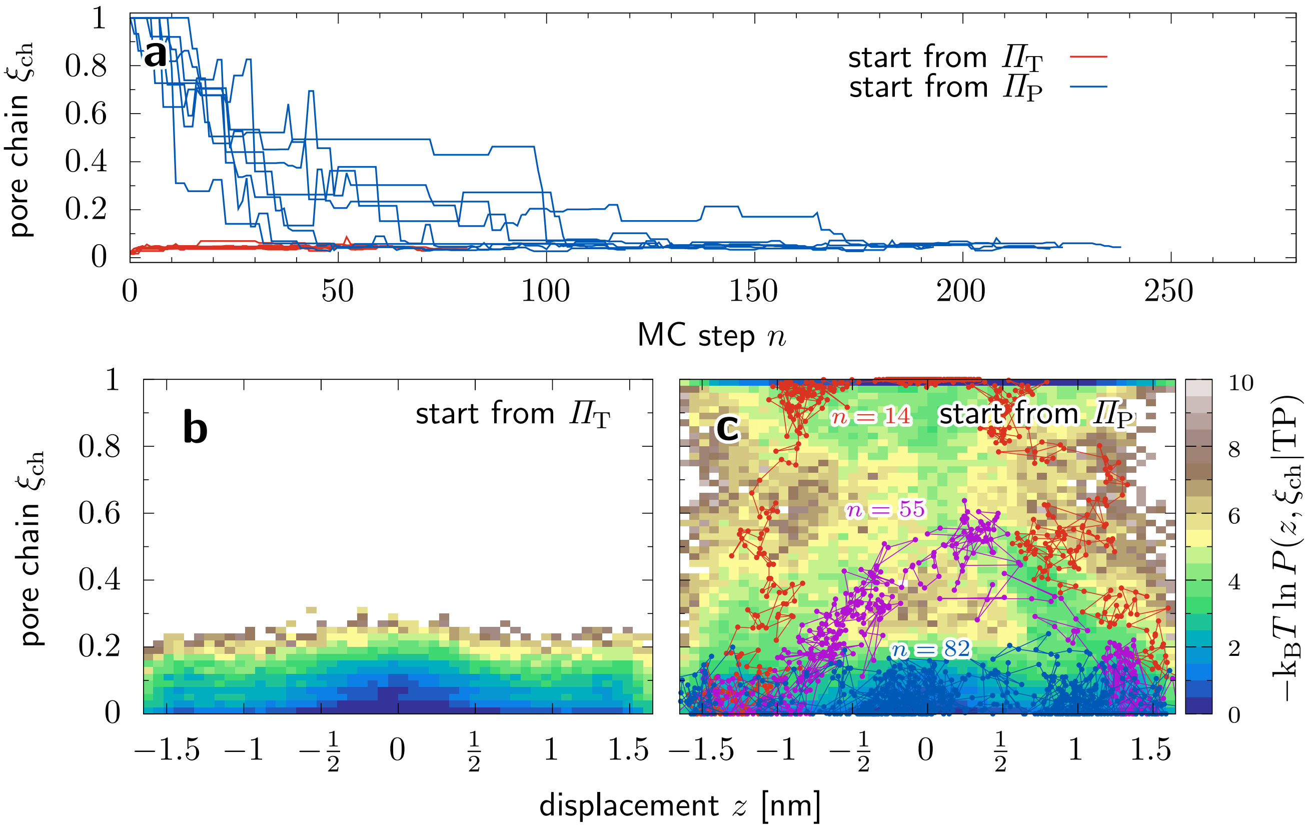

Supplementary Figure 12: Lipid flip-flop of cholesterol in a mammalian plasma membrane. a MC chains starting from a wet ($\mathit{\Pi}_\mathrm{P}$) and a dry mechanism ($\mathit{\Pi}_\mathrm{T}$), showing the time-averaged pore-chain coordinate $\hat{\xi}_\mathrm{ch}$ of Ref. 1. **b**,**c** TPE projected onto the transversal displacement z and ξ_ch in TP samplers starting from $\mathit{\Pi}_\mathrm{T}$ (**b**) and $\mathit{\Pi}_\mathrm{P}$ (**c**). In **c**, we highlight the trajectories of the sampler transitioning towards the tunnel mechanism with dots and lines.

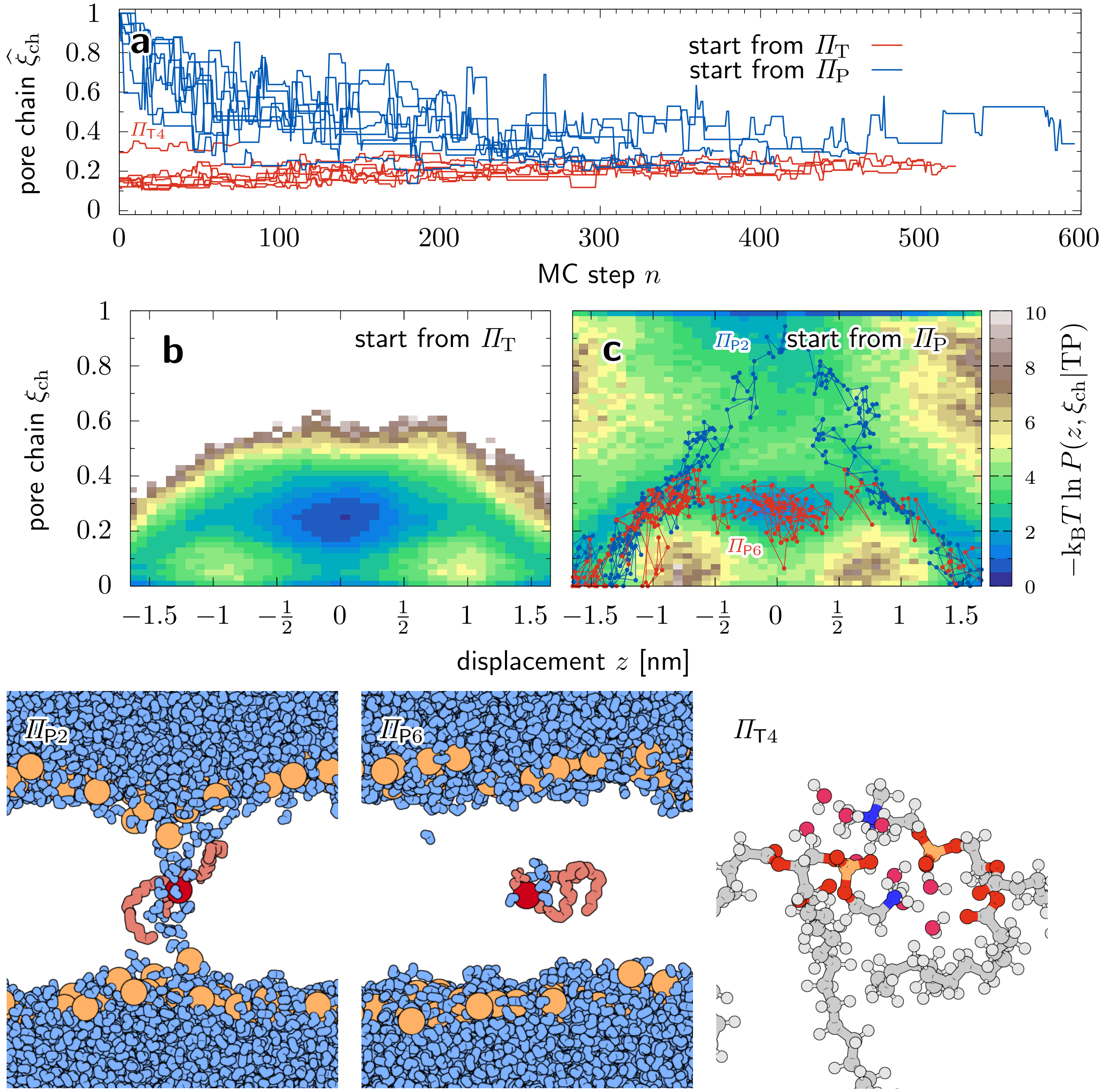

Supplementary Figure 13: Lipid flip-flop of PLPC lipids in a mammalian plasma membrane. a MC chains starting from a wet (Π_P) and a dry mechanism (Π_T), showing the time-averaged pore-chain coordinate $\hat{\xi}_\mathrm{ch}$ of Ref. 1. **b**,**c** TPE projected onto the transversal displacement z and ξ_ch in TP samplers starting from Π_T (**b**) and Π_P (**c**). Bottom three panels show exemplary snapshots of the last MC step of the annotated sampler (in Π_{T4}, see (**a**) on the left, the initial transition included two lipids, which consisted during the ~70 MC steps).

Supplementary Methods

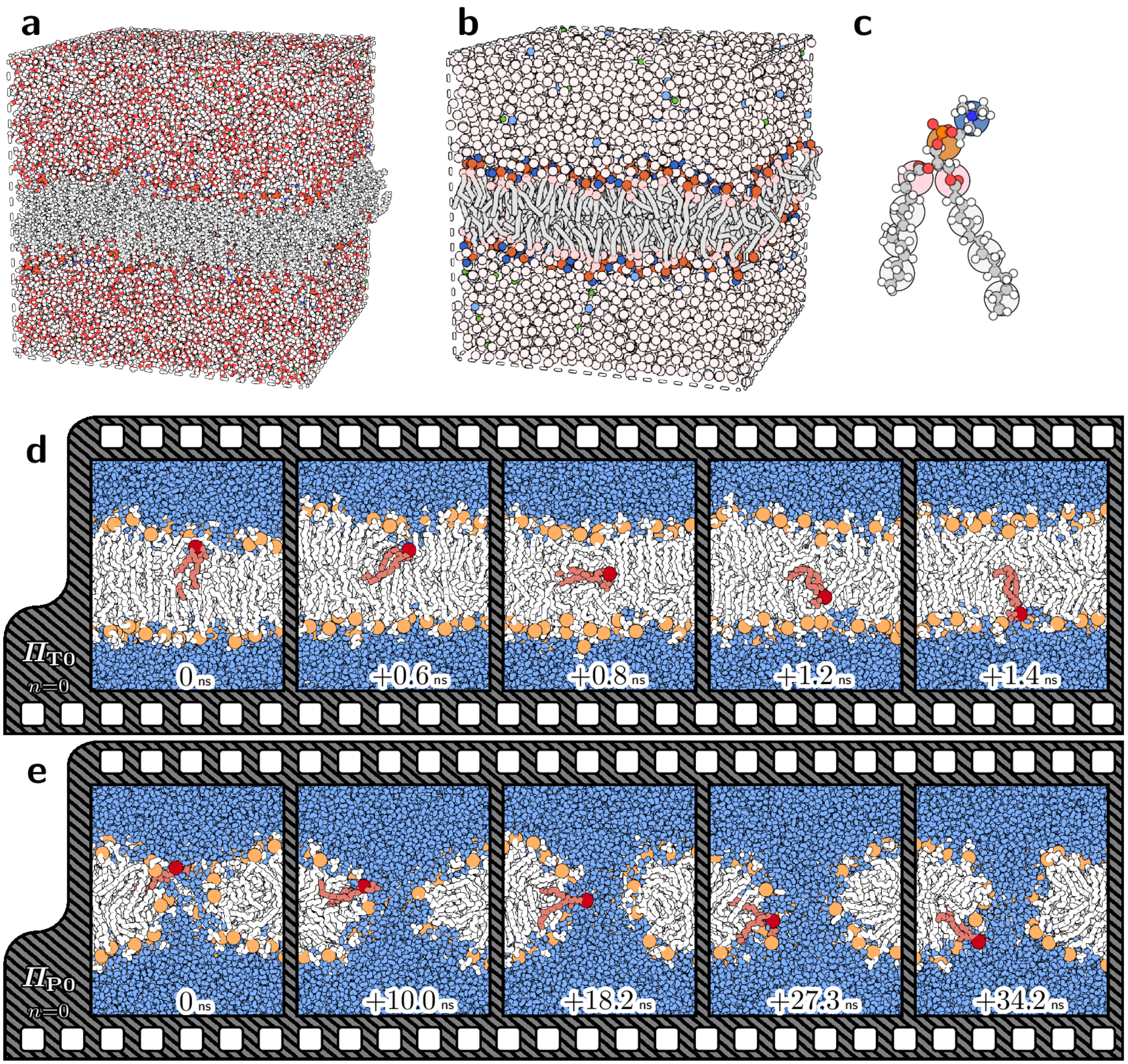

Supplementary Figure 14: Simulation setup. **a** All-atom and **b**,**c** Martini coarse-graining. System comprising 450 DMPC lipids at 310.15 K and 1 bar. **d** Exemplary flip-flop transition with water kept from entering the bilayer via flat-bottomed restraint in z. **e** The same with water pore kept open via cylindrical flat-bottomed restraint of the lipids, with increasing radius along the lipid tails.

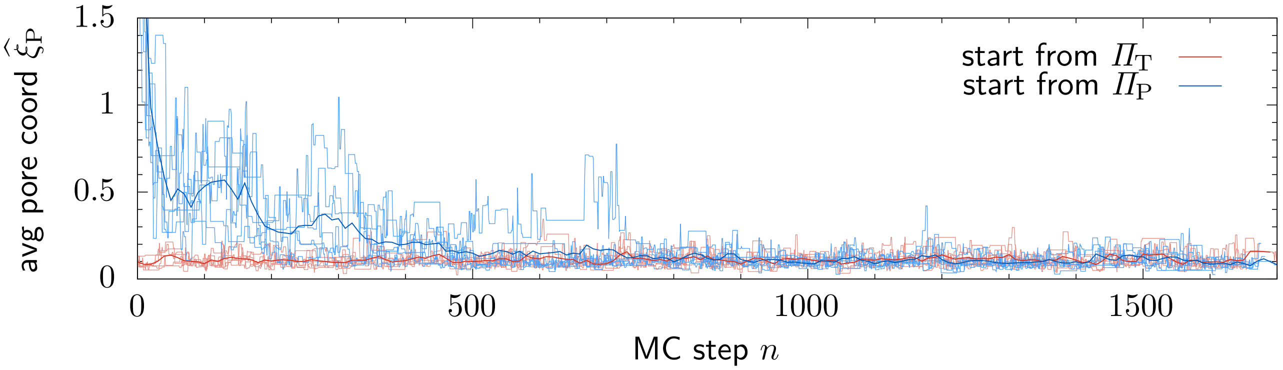

Supplementary Figure 15: Lipid flip-flop of Martini3 DMPC lipids with PME. MC chains starting from a wet (Π_P) and a dry mechanism (Π_T), showing the time-averaged pore coordinate $\hat{\xi}_\mathrm{P}$ adopted from Refs. 1,2.

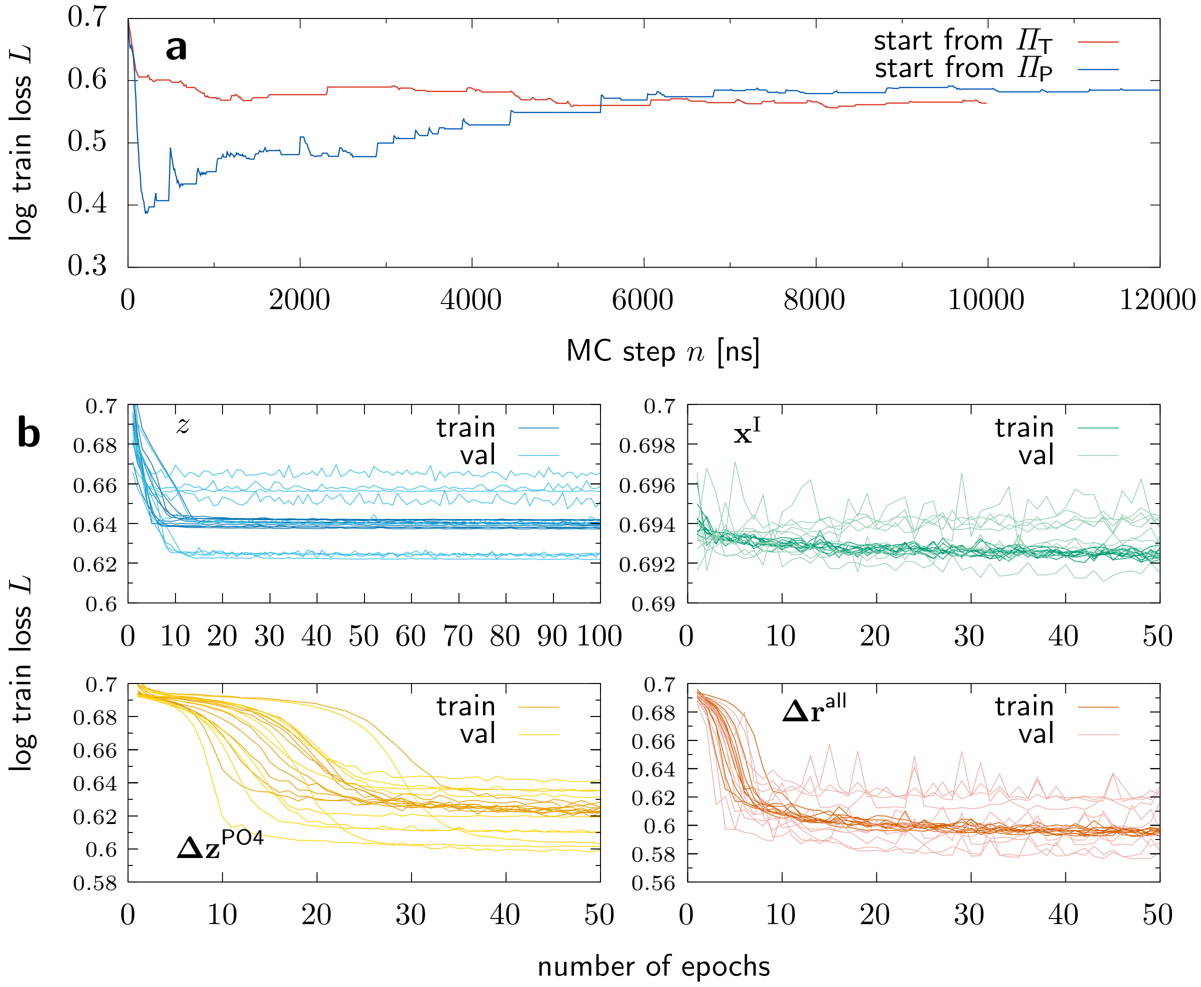

Supplementary Figure 16: Loss of Martini3 DMPC flip-flop models. **a** Loss during AIMMD runs of the initial (Supplementary Table 3) network models, individually trained on the MC chains starting from a wet (Π_P) and a dry mechanism (Π_T). **b** Loss of the models trained post-processing, comparing the four models highlighted in Fig. 2b of the main manuscript. Train loss (from 9 of the 10 folds used) is shown in dark colors, validation loss in faint colors.

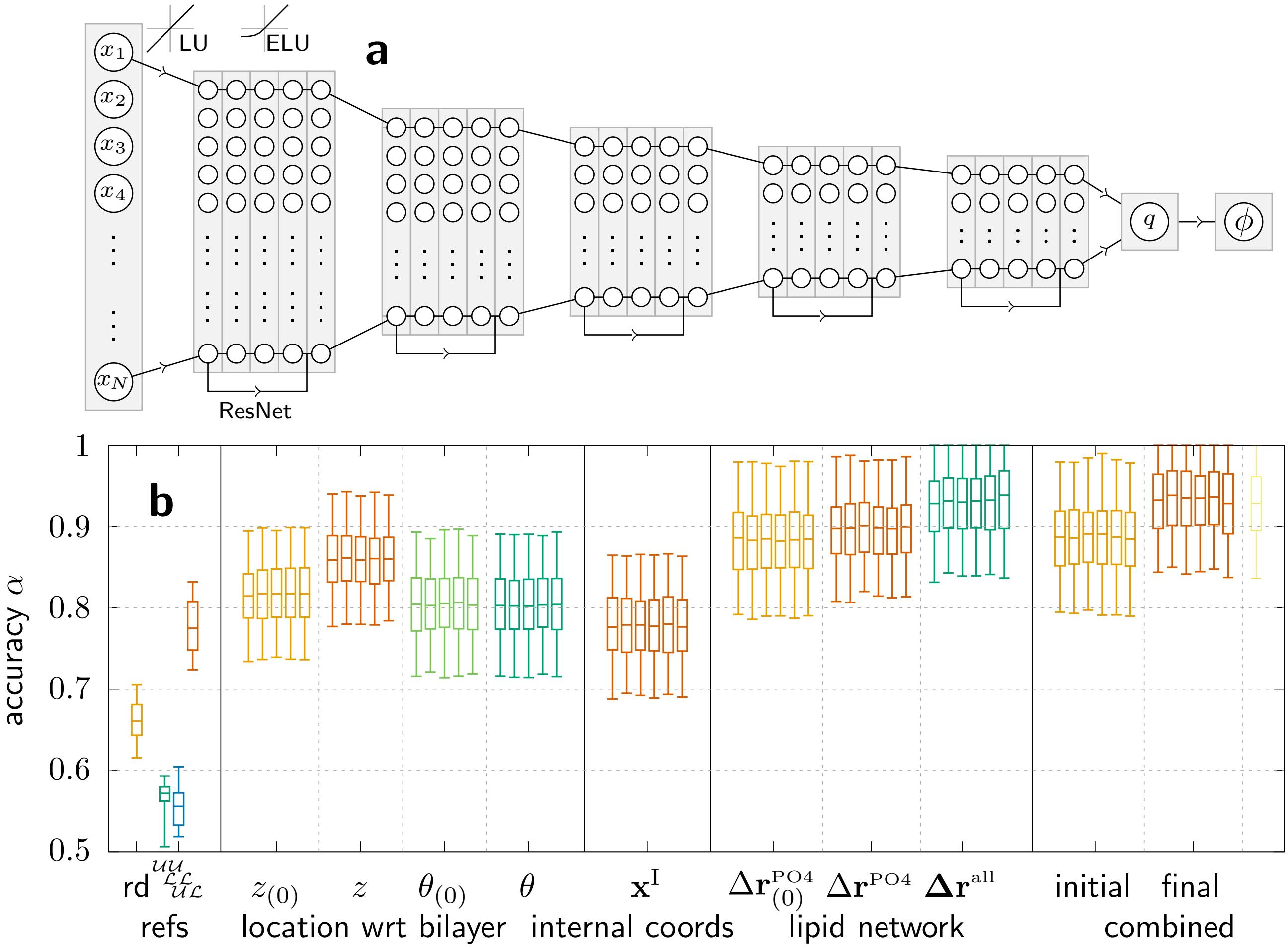

Supplementary Figure 17: Test of accuracy of various network models. **a** General network architecture used in this work. We feed in N input features $\mathbf{x}$, which are successively compressed via a linear unit (LU), after which a 5-layered ResNet[3] unit with exponential linear unit (ELU) activation function. The final one-dimensional output is then soft-maxed by an "expit" function. **b** Network accuracies. For reference, we guess the committor with uniform random numbers (rd) or always predict the same transition, $\phi_{\mathcal{UU}} = 0$, $\phi_{\mathcal{LL}} = 0$, or $\phi_{\mathcal{UL}} = 0.5$, with $\mathcal{U} \rightarrow$ upper and $\mathcal{L} \rightarrow$lower leaflet. We compare the transversal displacement w.r.t. the midplane defined by all PO4 beads ($z_{(0)}$, used by the original 66d network) with one defined by the lipid tail C beads, weighted by distances to probe (z). The tilt angle ($\theta_{(0)}$), defined by the z-axis is compared with a definition weighting the PO4 beads to calculate the membrane normal (θ). For the internal state we feed in a list of its distances, angles and dihedrals ($\mathbf{x}^{\mathrm{I}}$). The lipid network description compares the old definition of the relative x, y and z coordinates of the next-neighboring PO4 beads ($\Delta\mathbf{r}_{(0)}^{\mathrm{PO4}}$) with one sorting lipids in the upper and lower leaflet separately ($\Delta\mathbf{r}^{\mathrm{PO4}}$). We also take the first three lipids from the upper and lower leaflet and compute all distance combinations between all beads with the probe ($\Delta\mathbf{r}^{\mathrm{all}}$). Last, we combine these improved descriptors for a final committor model (see Supplementary Table 5). We estimate the accuracy, Eq. (2), by bootstrapping: With 10 folds, we split the data into 0.9 training and 0.1 validation (always leaving one of the ten samplers out), then train the network using a number of epochs, which minimizes the loss of the validation set. We then make 100 times a bootstrap sample from the validation set with repetition and calculate α. Boxes show the median and 50% of the data, whiskers 95%. Boxes of same colors show same input features but different bottleneck architectures. The last box on the right shows the final network, which uses an additional L2 regularization term.

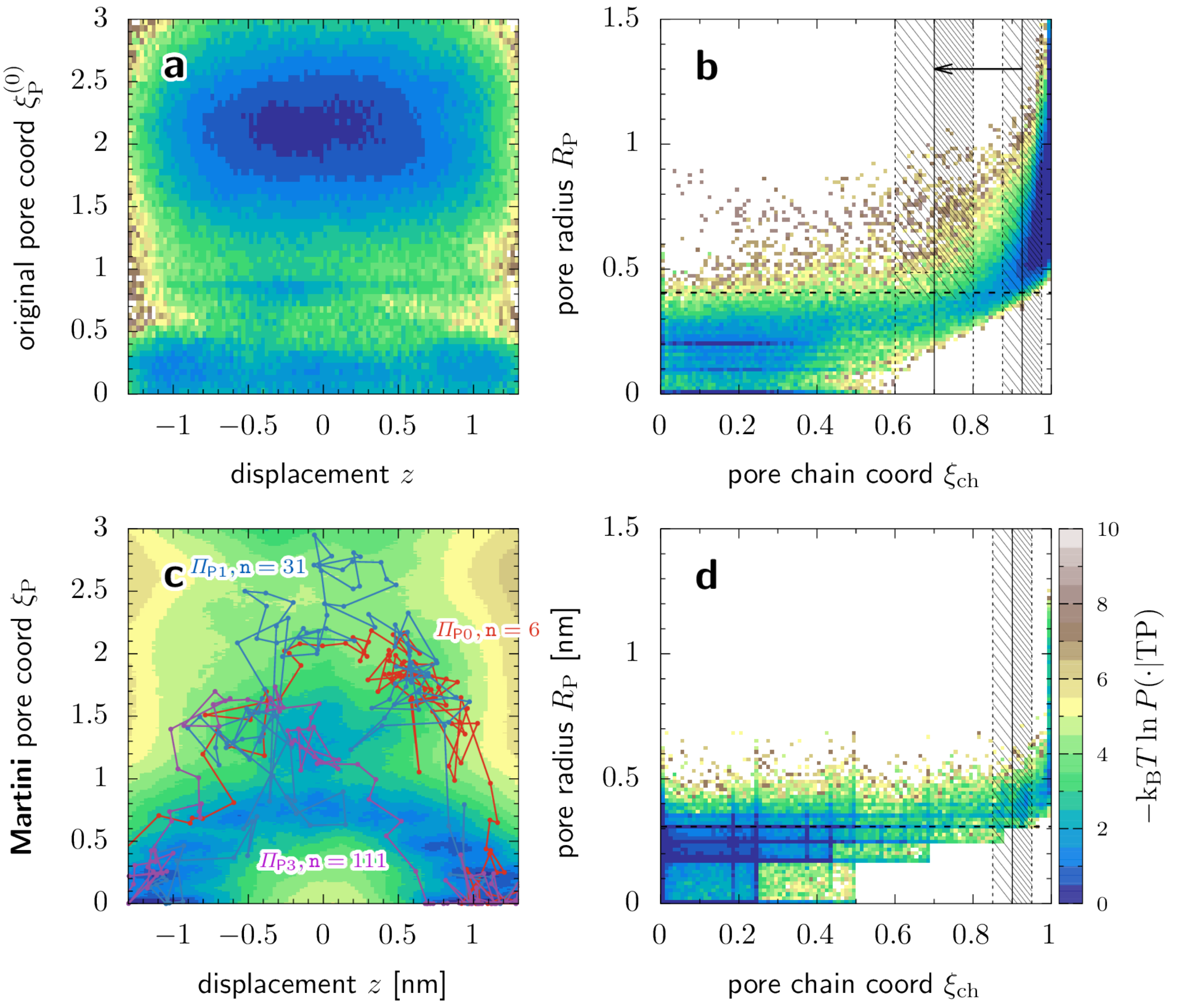

Supplementary Figure 18: Calibration of the pore reaction coordinate. **a,b** TPS data of the all-atom Charmm36 DMPC lipids, projected onto coordinates describing the water pore. **a** The original definition in Ref. 2, $\xi_P^{(0)}$, shows an artificial meta-stable state due to its definition. **b** TPE projected onto the constituting coordinates, showing the old "switch" value $\xi_{ch}^{s} = 0.925$ up to which only the chain coordinate ξ_{ch} is contributing to ξ_P, and the pore radius at that threshold, $R_0 = 0.405$. We instead use

$$\xi_P(\mathbf{r}) := \xi_{ch}(\mathbf{r}) + H_\varepsilon[\xi_{ch}(\mathbf{r}) - \xi_{ch}^{s}] \times \Theta_\delta\left[\frac{R_P(r)-R_0}{R_0}\right], \quad \Theta_\delta(x) := \begin{cases} \delta e^{\frac{x-\delta}{\delta}} & \text{for } x < \delta, \\ x & \text{else.} \end{cases}$$

with $\xi_{ch}^{s} = 0.7$, $R_0 = 0.405$ nm, $\varepsilon = 0.1$ and $\delta = 0.2$, which removes the artifact. (**c**,**d**) TPS data of the Martini DMPC lipids, projected onto coordinates describing the water pore. We use the data starting from Π_P to calibrate the parameters for ξ_{ch}, R_P and ξ_P to be able to describe the observed water defects: 4 subdivisions, $z_{mem} = 1.8$ nm, $R_{cyl} = 1.0$ nm, $D = 1.2$ nm, $\xi_{ch}^{s} = 0.9$, $R_0 = 0.38$ nm. (**c**) k-NN density of all data showing also the initial shrinking and collapse. We show exemplary trajectories at that point in the MC chain. (**d**) Data projected onto the constituting ξ_{ch} and R_P. The sharp peaks in the density show how many water beads are in the midplane, motivating to use a small number of subdivisions. We then choose the switching region at the point where R_P starts to rise above its plateau for small ξ_{ch}.

Supplementary Tables

Supplementary Table 1. Architectures of tested networks. Dropout during training shown in parentheses above arrows. Each arrow represents a linear compression, after which follows a ResNet unit, see Fig. S13a. Number of training epochs were chosen at the minimum of the validation loss. We try out all architectures for the location w.r.t. the bilayer individually. For the final model, we use L2 regularization to enable a large number of epochs.

List of networks		
System	Model	Description
Reference	rand(om)	Committor guessed randomly (uniform random numbers)
Martini DMPC	AIMMD model	Model trained during the AIMMD iterations. Uses features<br>$z_{(0)}$, $\theta_{(0)}$, R_G, $\sigma_{\mathcal{U}}$, $\sigma_{\mathcal{L}}$, $\Delta\mathbf{r}^{\mathrm{PO4}}_{(0)}$, N^{NN}, N^{NN}, N^{W} (see Supplementary Table 3)<br>with network $68 \xrightarrow{0.22} 49 \xrightarrow{0.16} 35 \xrightarrow{0.12} 26 \xrightarrow{0.08} 18 \xrightarrow{0.06} 13 \xrightarrow{0.04} 10 \longrightarrow 1$.
	z	Using only the midplane displacement defined in Supplementary Table 5, with architecture $1 \longrightarrow 10 \longrightarrow 1$.
	$\Delta\mathbf{r}^{\mathrm{PO4}}$	Using the coordinates of the 10 + 10 nearest neighboring lipids from the upper/lower leaflet, respectively. $60 \xrightarrow{0.21} 41 \xrightarrow{0.15} 29 \xrightarrow{0.10} 20 \xrightarrow{0.07} 14 \xrightarrow{0.05} 10 \longrightarrow 1$.
	$\Delta\mathbf{r}^{\mathrm{all}}$	Using all distances between the 10 beads of the 3+3 nearest neighbors.<br>$600 \xrightarrow{0.13} 264 \xrightarrow{0.06} 116 \xrightarrow{0.03} 51 \xrightarrow{0.01} 22 \xrightarrow{0.005} 10 \longrightarrow 1$.
	"final" model	Using a combined list of improved features, $\mathbf{x} =$<br>$(z, \theta, d_{\mathrm{C3A-C3B}}, d_{\mathrm{C2A-C2B}}, \theta_{\mathrm{C3A-GL1-C3B}}, \theta_{\mathrm{PO4-GL1-C1A}}, \theta_{\mathrm{PO4-GL2-C1B}}, \Delta\mathbf{r}^{\mathrm{PO4}}, \Delta\mathbf{r}^{\mathrm{all}})$<br>(see Supplementary Table 5). $667 \longrightarrow 287 \longrightarrow 124 \longrightarrow 53 \longrightarrow 23 \longrightarrow 10 \longrightarrow 1$.
	linear model	Using $\mathbf{x} \cdot \mathbf{v}$ (plus z and θ) as inputs, $3 \longrightarrow 5 \longrightarrow 10 \longrightarrow 10 \longrightarrow 1$.
Charmm36 DMPC lipid flip-flop	AIMMD model	Model trained during the AIMMD iterations. Uses features<br>$z_{(0)}$, $\theta_{(0)}$, R_G, $\sigma_{\mathcal{U}}$, $\sigma_{\mathcal{L}}$, $\Delta\mathbf{r}^{\mathrm{PO4}}_{(0)}$, N^{NN}, N^{NN}, N^{W} (see Supplementary Table 3)<br>with network $68 \xrightarrow{0.22} 49 \xrightarrow{0.16} 35 \xrightarrow{0.12} 26 \xrightarrow{0.08} 18 \xrightarrow{0.06} 13 \xrightarrow{0.04} 10 \longrightarrow 1$.
	z	Using only the midplane displacement defined in Supplementary Table 5, with architecture $1 \longrightarrow 10 \longrightarrow 1$. Either trained on all data ($\Pi_{\mathrm{T}} + \Pi_{\mathrm{P}}$) or only on the pore transitions (Π_{P})
	$\Delta\mathbf{r}^{\mathrm{all}}$	Using all distances between 10 atoms of the 3+3 nearest neighbors, corresponding to the 10 beads of the Martini model: N, P, O31, O32, C24, C34, C28, C38, C212 and C312. Network encodes as $600 \xrightarrow{0.13} 264 \xrightarrow{0.06} 116 \xrightarrow{0.03} 51 \xrightarrow{0.01} 22 \xrightarrow{0.005} 10 \longrightarrow 1$.<br>$29 \xrightarrow{0.21} 20 \xrightarrow{0.15} 14 \xrightarrow{0.10} 10 \longrightarrow 1$
	"final" model	Using a combined list of improved features, $\mathbf{x} = (z, \theta, \xi_{\mathrm{P}}, \Delta\mathbf{r}^{\mathrm{PO4}}, \Delta\mathbf{r}^{\mathrm{all}})$ (see Supplementary Table 5). $663 \longrightarrow 287 \longrightarrow 124 \longrightarrow 53 \longrightarrow 23 \longrightarrow 10 \longrightarrow 1$.
	linear model	Using $\mathbf{x} \cdot \mathbf{v}$ (plus z and ξ_{P}) as inputs, $3 \longrightarrow 5 \longrightarrow 10 \longrightarrow 10 \longrightarrow 1$.
Charmm36 DMPC pore formation	AIMMD model	Model trained during the AIMMD iterations. Uses features introduced by Hub/Awasthi and Bubnis/Grubmüller (see Supplementary Table 4). with network<br>$147 \xrightarrow{0.18} 85 \xrightarrow{0.10} 50 \xrightarrow{0.06} 29 \xrightarrow{0.03} 17 \xrightarrow{0.02} 10 \longrightarrow 1$.
	ξ_{P}	Using only the pore reaction coordinate defined in Ref. 1.<br>With architecture $1 \longrightarrow 10 \longrightarrow 1$.
	z_1	Using only the z displacement closest to the nucleation core. $1 \longrightarrow 10 \longrightarrow 1$.
	$\Delta z^{\mathrm{max}}_{NP1-4}$	Using the maximum distance of the 4th nearest neighbors between N or P atoms, see Ref 4. With architecture $1 \longrightarrow 10 \longrightarrow 1$.
Charmm36 DSPC lipid flip-flop	AIMMD model	Model trained during the AIMMD iterations. Uses features z, θ,<br>ξ_{P}, $\Delta\mathbf{r}^{\mathrm{PO4}}$, $\Delta\mathbf{r}^{\mathrm{all}}$ (see Supplementary Table 5), adding C216 and C316 as atoms.<br>with network $927 \xrightarrow{0.12} 374 \xrightarrow{0.05} 151 \xrightarrow{0.02} 61 \xrightarrow{0.008} 24 \xrightarrow{0.003} 10 \longrightarrow 1$.

Charmm36 Mammalian plasma membrane	AIMMD model or CHOL flip-flop	Model trained during the AIMMD iterations. Uses only features z and $\Delta\mathbf{z}^{\mathrm{PO4/O3}}$ of the 10+10 nearest neighboring heads (O3 is the bead of cholesterol), $21 \xrightarrow{0.26} 18 \xrightarrow{0.22} 15 \xrightarrow{0.19} 13 \xrightarrow{0.17} 11 \xrightarrow{0.14} 10 \longrightarrow 1$.
	AIMMD model or PLPC flip-flop	Model trained during the AIMMD iterations. Uses only features z and $\Delta\mathbf{z}^{\mathrm{PO4/O3}}$ of the 10+10 nearest neighboring heads (O3 is the bead of cholesterol), $21 \xrightarrow{0.26} 18 \xrightarrow{0.22} 15 \xrightarrow{0.19} 13 \xrightarrow{0.17} 11 \xrightarrow{0.14} 10 \longrightarrow 1$.

Supplementary Table 2. Lipid composition of mammalian plasma membrane adopted from Ref. 5.

Plasma membrane composition		
Name (Charmm GUI)	Details	Number (UL/LL)
POPC	PC(16:0/18:1(9Z))	32/14
PLPC	PC(16:0/18:2(9Z,12Z))	44/22
PAPE	PE(16:0/20:4(5Z,8Z,11Z,14Z))	6/24
POPE	PE(16:0/18:1(9Z))	6/28
POPI	PI(16:0/18:1(9Z))	0/10
PAPS	PS(16:0/20:4(5Z,8Z,11Z,14Z))	0/22
POPA	PO(16:0/18:1(9Z))	0/2
SSM	SM(d18:1/18:0)	22/10
NSM	SM(d18:1/24:1)	22/10
CMH/GLPA (CER160+BGAL)	GlcCer(d18:1/16:0)	8/0
CHL1	Cholesterol	74/58
TIP3	Water	27088
SOD	Sodium ions	108
CLA	Chlorine ions	74

Supplementary Table 3. Input features used for the committor model of lipid flip-flop trained during AIMMD.

Initial descriptors		
Category	Name	Description
location w.r.t. midplane	$z_{(0)}$	Transversal displacement. We define the mid-plane as the center of all P (PO_4) atoms. z is the distance of the probe's P (PO_4) in z-direction of the box w.r.t. that midplane.
	$\theta_{(0)}$	Tilt angle. We use the average distance vector of each atom of the probe to its center of mass and calculate the scalar product with the z-axis.
internal coordinates	R_G	Radius of gyration. Mass-weighted average of distances of each atom to the center of mass.
membrane	σ_u	Upper leaflet displacement. σ_u is calculated as the standard deviation of z positions of P (PO4) atoms of the upper leaflet w.r.t. to their center.

	$\sigma_{\mathcal{L}}$	Lower leaflet displacement. $\sigma_{\mathcal{L}}$ is calculated as the standard deviation of z positions of P (PO4) atoms of the lower leaflet w.r.t. to their center.
lipid network	$\Delta\mathbf{x}_{(0)}^{\mathrm{PO4}}$, $\Delta\mathbf{y}_{(0)}^{\mathrm{PO4}}$, $\Delta\mathbf{z}_{(0)}^{\mathrm{PO4}}$	Distance to ith NN lipid. On every frame of the trajectory, we sort the P (PO4) atoms by distance to the probe P (PO4). We then only keep the 20 nearest neighbors, but use all 3 signed displacements.
water	N^{W}	Number of water molecules next to probe. We use indicator functions of Ref. 6, $N^{NN} = \sum_i \frac{1}{2}\left[1 - \tanh\left(a(r_i - r_0)\right)\right]$, where r_i is the distance of the probes P (PO_4) to the ith O (W) water atom. We use $a = 50$, $r_0 = 0.43$ ($a = 30$, $r_0 = 0.63$ Martini), corresponding to the first maximum of the RDF.
	N^{NNN}	Number of water molecules close to probe. We use $a = 10$, $r_0 = 0.78$ ($a = 8$, $r_0 = 1.2$ Martini), corresponding to the first maximum of the RDF.
	N^{W}	Number of water molecules in pore. We use the same indicator, but only with (absolute) distance in z-direction, with $a = 10$, $r_0 = 0.5$, corresponding to the width of the membrane.

Supplementary Table 4. Input features used for the committor model of pore nucleation trained during AIMMD.

Initial descriptors		
Category	Name	Description
Hub / Awasthi	ξ_{ch}	Chain reaction coordinate as detailed in Ref. 1.
	R_{P}	Pore radius as detailed in Ref. 2
	ξ_{P}	Pore reaction coordinate as detailed in Ref. 2
Bubnis / Grubmueller	$\Delta\mathbf{r}_1^X$, $\Delta\mathbf{r}_2^X$, $\Delta\mathbf{r}_3^X$	Isotropic distance of closest, next, and next-to-next atom of group X to the pore center. X= all water oxygens, all phosphorus atoms, all phosphorus+nitrogen, all phosphorus+nitrogen+water oxygen, all lipid tail's carbons, and all carbons, as detailed in Ref. 4. The pore center we compute as detailed in Ref. 1.
	$\Delta\boldsymbol{\rho}_1^X$, $\Delta\boldsymbol{\rho}_2^X$, $\Delta\boldsymbol{\rho}_3^X$	Lateral distance of the same.
	$\Delta\mathbf{z}_1^X$, $\Delta\mathbf{z}_2^X$, $\Delta\mathbf{z}_3^X$	Axial distance of the closest atoms.
	$\Delta\mathbf{r}_{1-2}^X$, $\Delta\mathbf{r}_{1-3}^X$, $\Delta\mathbf{r}_{1-4}^X$, $\Delta\mathbf{r}_{1-5}^X$, $\Delta\mathbf{r}_{1-10}^X$	Isotropic distance averaged over the closest atoms as indicated, i.e., over the 2, 3, 4, 5 and 10 atoms closest to the nucleation center.
	$\Delta\boldsymbol{\rho}_{1-2}^X$, $\Delta\boldsymbol{\rho}_{1-3}^X$, $\Delta\boldsymbol{\rho}_{1-4}^X$, $\Delta\boldsymbol{\rho}_{1-5}^X$, $\Delta\boldsymbol{\rho}_{1-10}^X$	Lateral distance of the same.
	$\Delta\mathbf{z}_{X1-2}^{\mathrm{max}}$, $\Delta\mathbf{z}_{X1-3}^{\mathrm{max}}$, $\Delta\mathbf{z}_{X1-4}^{\mathrm{max}}$, $\Delta\mathbf{z}_{X1-5}^{\mathrm{max}}$, $\Delta\mathbf{z}_{X1-10}^{\mathrm{max}}$	The closest 100 atoms to the center are sorted by their z-position. Then, we loop over pairs of 2, 3, 4, 5 and 10 atoms, respectively, and calculate the average distance to their average z-position. The "depletion" of atoms is then the respective maximum.

Supplementary Table 5. Input features used for the committor models trained post-simulation.

Additional descriptors – Martini Name Description		
Category	Name	Description
position w.r.t. midplane	z	We define the mid-plane via all lipid tail carbon beads. We weigh them by their distance to the probe: We use the sigmoid of Ref. [7], $w_i = \left[1 + \left(2^{a/b} - 1\right)(\Delta r/s)^a\right]^{-b/a}$, with $a = 8$, $b = 3$, $\sigma = 2$ nm, to weigh lipids with distance $\Delta r < \sigma$ more.
	θ	Instead of the z-axis, we use the distance vector spanned by the center of PO4 beads of the upper leaflet and the lower leaflet. We weight these atoms again by distance to the probes P atom, as above.
internal coordinates	$d_{\mathrm{GL1-GL2}}$, $d_{\mathrm{C1A-C1B}}$, $d_{\mathrm{C2A-C2B}}$, $d_{\mathrm{C3A-C3B}}$, $d_{\mathrm{NC3-C1A}}$, $d_{\mathrm{NC3-C2A}}$, $d_{\mathrm{NC3-C3A}}$, $d_{\mathrm{NC3-C1B}}$, $d_{\mathrm{NC3-C2B}}$, $d_{\mathrm{NC3-C3B}}$	Distance between beads of lipid probe.
	$\theta_{\mathrm{NC3-PO4-GL1}}$, $\theta_{\mathrm{NC3-PO4-GL2}}$, $\theta_{\mathrm{GL1-PO4-GL2}}$, $\theta_{\mathrm{C1A-PO4-C1B}}$, $\theta_{\mathrm{C2A-PO4-C2B}}$, $\theta_{\mathrm{C3A-PO4-C3B}}$, $\theta_{\mathrm{PO4-GL1-C1A}}$, $\theta_{\mathrm{GL1-C1A-C2A}}$, $\theta_{\mathrm{C1A-C2A-C3A}}$, $\theta_{\mathrm{PO4-GL2-C1B}}$, $\theta_{\mathrm{GL2-C1B-C2B}}$, $\theta_{\mathrm{C1B-C2B-C3B}}$	Angles between beads of lipid probe.
	$\varphi_{\mathrm{NC3-PO4-GL1-GL2}}$, $\varphi_{\mathrm{NC3-PO4-GL1-C1A}}$, $\varphi_{\mathrm{PO4-GL1-C1A-C2A}}$, $\varphi_{\mathrm{GL1-C1A-C2A-C3A}}$, $\varphi_{\mathrm{NC3-PO4-GL2-C1B}}$, $\varphi_{\mathrm{PO4-GL2-C1B-C2B}}$, $\varphi_{\mathrm{GL2-C1B-C2B-C3B}}$	Dihedral between beads of lipid probe.
lipid network	$\Delta\mathbf{x}^{\mathrm{PO4}}$, $\Delta\mathbf{y}^{\mathrm{PO4}}$, $\Delta\mathbf{z}^{\mathrm{PO4}}$	Lipids PO4 displacement sorted by a fixed reference. We calculate their distance to the probes PO4 over time to measure their importance weight $w_i(\Delta\mathbf{r})$ with $a = 20$, $b = 8$ and $\sigma = 0.7$. We then take the time average to sort by highest importance, and save the 10 most important lipids of the lower and upper leaflet, respectively.
	$\Delta\mathbf{r}^{\mathrm{all}}$	We use the above ranking to calculate the distance matrix between all 10 beads of the probe and the ith ranked lipid. We save the three most important (see above) lipids of the lower and upper leaflet, respectively.

Supplementary References

1 Hub, J. S. & Awasthi, N. Probing a Continuous Polar Defect: A Reaction Coordinate for Pore Formation in Lipid Membranes. *J. Chem. Theory Comput.* **13**, 2352-2366 (2017). https://doi.org/10.1021/acs.jctc.7b00106

2 Hub, J. S. Joint Reaction Coordinate for Computing the Free-Energy Landscape of Pore Nucleation and Pore Expansion in Lipid Membranes. *Journal of Chemical Theory and Computation* **17**, 1229-1239 (2021). https://doi.org/10.1021/acs.jctc.0c01134

3 He, K., Zhang, X., Ren, S. & Sun, J. in *2016 IEEE Conference on Computer Vision and Pattern Recognition (CVPR).* 770-778.

4 Bubnis, G. & Grubmüller, H. Sequential Water and Headgroup Merger: Membrane Poration Paths and Energetics from MD Simulations. *Biophys. J.* **119**, 2418-2430 (2020). https://doi.org/10.1016/j.bpj.2020.10.037

5 Pogozheva, I. D. *et al.* Comparative Molecular Dynamics Simulation Studies of Realistic Eukaryotic, Prokaryotic, and Archaeal Membranes. *Journal of Chemical Information and Modeling* **62**, 1036-1051 (2022). https://doi.org/10.1021/acs.jcim.1c01514

6 Mullen, R. G., Shea, J.-E. & Peters, B. Transmission Coefficients, Committors, and Solvent Coordinates in Ion-Pair Dissociation. *J. Chem. Theory Comput.* **10**, 659-667 (2014). https://doi.org/10.1021/ct4009798

7 Tribello, G. A., Ceriotti, M. & Parrinello, M. Using sketch-map coordinates to analyze and bias molecular dynamics simulations. *PNAS* **109**, 5196-5201 (2012). https://doi.org/10.1073/pnas.1201152109
